# IRF3 is an important molecule in the UII/UT system and mediates immune inflammatory injury in acute liver failure

**DOI:** 10.18632/oncotarget.10717

**Published:** 2016-07-19

**Authors:** Liang-ming Liu, Wen-juan Tu, Tong Zhu, Xiao-ting Wang, Zhi-li Tan, Huan Zhong, De-yong Gao, Dong-yu Liang

**Affiliations:** ^1^ Department of Hepatology, Songjiang Hospital Affiliated to the First People's Hospital Shanghai Jiaotong University, Shanghai, China

**Keywords:** IRF3, urotensin II, acute liver failure, immune-mediated inflammation, Kupffer cells, Immunology and Microbiology Section, Immune response, Immunity

## Abstract

The urotensin II/urotensin receptor (UII/UT) system can mediate inflammatory liver injury in acute liver failure (ALF); however; the related mechanism is not clear. In this study, we confirmed that lipopolysaccharide/D-galactosamine (LPS/D-GalN) induced up-regulation of liver interferon regulatory factor 3 (IRF3) in ALF mice, whereas the UT antagonist urantide inhibited the up-regulated liver IRF3. LPS stimulation induced IRF3 transcription and nuclear translocation and promoted the secretion of interleukin-6 (IL-6), interferon (IFN)-β, and IFN-γ in Kupffer cells (KCs); these effects in LPS-stimulated KCs were inhibited by urantide. Knockdown of IRF3 using an adenovirus expressing an IRF3 shRNA inhibited IFN-β transcription and secretion as well as tumor necrosis factor (TNF)-α and IL-1β secretion from LPS-stimulated KCs; additionally, IL-10 transcription and secretion were promoted in response to LPS. However, LPS-stimulated TNF-α and IL-1β mRNA was not affected in the KCs. The IRF3 shRNA also did not have a significant effect on the NF-κB p65 subunit and p38MAPK protein phosphorylation levels in the nuclei of LPS-stimulated KCs. Therefore, IRF3 expression and activation depended on the signal transduction of the UII/UT system, and played important roles in UII/UT-mediated immune inflammatory injury in the liver but did not affect NF-κB and p38 MAPK activity.

## INTRODUCTION

Acute liver failure (ALF) is a liver tissue injury disease that has an immune-mediated inflammatory reaction as its main feature [1]. Pro-inflammatory cytokines, especially tumor necrosis factor (TNF)-α, play critical roles in the occurrence and development of liver inflammation in ALF [2]. Recently, the vasoactive peptide substance urotensin II (UII) was shown to possess significant inflammatory activity.

UII is a cyclic polypeptide molecule that contains 11 amino acids. UII was first isolated from the tail bone of a bony fish; then, it was shown to be extensively distributed in mammals including humans in the cardiovascular system, the central nervous system, the lung, the kidney, the spleen, the pituitary gland, the adrenal gland, the stomach, the pancreas, the ovary, and the liver [3-5]. UII has many physiological and pathological activities and can regulate endocrine, cardiovascular, kidney, and immune functions [6]. UII molecule signaling is primarily transduced by the specific orphan G protein-coupled receptor (i.e., the urotensin receptor or UII receptor, which is primarily abbreviated UT) [7]. Studies showed that UII could function with UT to promote the aggregation of monocytes and macrophages at inflammatory injury sites and to promote the expression of adhesion molecules and chemokines and the release of inflammatory cytokines [8-10]. Patients with inflammatory vascular injury diseases such as atherosclerosis, chronic inflammatory cardiac injury diseases, immune-mediated inflammatory kidney injury diseases, and chronic inflammatory liver injury diseases all have high levels of UII secretion in the blood [11-14]. UII/UT expression is also significantly increased in the livers of ALF patients [15]. Our recent study showed that LPS/D-GalN induced an abnormal elevation of UII expression and secretion and UT expression in the livers of ALF experimental mice. The application of the UT-specific antagonist urantide significantly inhibited UII/UT expression in the mouse liver, relieved inflammatory injury of the liver tissues, and down-regulated the levels of the pro-inflammatory cytokines TNF-α and IL-1β [16]. These results suggested that the UII/UT signaling system mediated LPS/D-GalN-induced inflammatory liver injury in ALF.

In ALF, Kupffer cells (KCs) are the primary cells that express UII/UT [15, 16]. KCs are tissue resident macrophages in the liver. As the major innate immune cells in the liver, KCs are the first line of defense and target pathogens from the intestinal tract and their released toxins [17]. Exogenous [18] and endogenous [17] injury stimulations can induce inflammatory activation of KCs, resulting in the cascade release of pro-inflammatory cytokines that causes acute liver injury or liver failure [19]. The functions of pathogens or toxins (i.e., LPS) can activate the pattern recognition receptor Toll-like receptor 4 (TLR4) on the KC surface. Through adaptor proteins, TLR4 activates two important cellular signaling pathways [the MyD88-dependent and TRIF-dependent (or MyD88-independent) pathways] [20]. After activation, the MyD88-dependent pathway can induce the expression and release of pro-inflammatory cytokines such as TNF-α, interleukin (IL)-1β, and IL-6 through the p38 mitogen-activated protein kinase (p38 MAPK) and nuclear factor-κB (NF-κB) signaling pathways [20]. TRIF signaling can induce the production of type 1 interferons such as IFN-β through the activation of interferon regulatory factor 3 (IRF3) to exert the innate immune response [19]. Our previous study showed that the UII/UT system activated the NF-κB and MAPK inflammatory signaling pathways [20]. However, whether this signaling system affects the TRIF-dependent (or MyD88-independent) IRF3 innate immune pathway is currently unclear.

IRF3 is an IRF family member. Under normal conditions, IRF3 is constitutively expressed in its monomer inactive form in a variety of innate immune cells, including macrophages and dendritic cells, where it exerts an effect through its transcription factor function. Upon exogenous stimulation, IRF3 can be activated in the cells to form homodimers or heterodimers. Activated IRF3 enters the nucleus to induce IFN-β transcription and exerts an innate anti-viral and anti-bacterial immune effect [21]. Viruses, bacteria, and parasites all activate IRF3 to induce innate immune reactions [22-24]. During pathogenic microbe infection, the increase in the expression of the IRF3 transcription factor in macrophages has a close positive correlation with the innate immune response against pathogenic microbes [25]. During liver tissue injury, immunity and inflammation are usually closely associated; however, the effect of IRF3 activation in KCs on inflammatory signaling is not clear.

The main purpose of this study was to investigate the influence of the UII/UT system on IRF3 signaling and the function of IRF3 in inflammatory reaction. This study will help elucidate the inflammatory effects of the IRF3 molecule and the molecular mechanism underlying the acute inflammatory liver injury mediated by the UII/UT system.

## RESULTS

### Urantide down-regulated liver IRF3 expression in LPS/D-GalN-induced ALF

**Figure 1:**
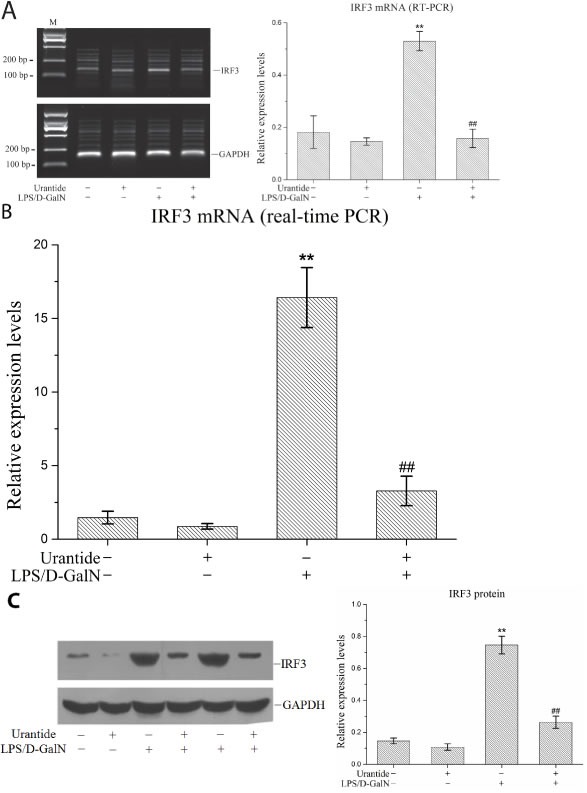
urantide downregulates IRF3 expression induced by LPS/D-GalN in liver in ALF mice Mice were treated with urantide or vehicle 0.5 h before LPS/D-GalN injection. **A**. IRF3 mRNA expression in liver using RT-PCR assay. Left panel shows a representative ethidium bromide-stained gel of RT-PCR products, and right shows relative expression levels of IRF3 mRNA in liver after normalization to GAPDH. Data represent means ± SD (n = 6 each group). **B**. IRF3 mRNA expression in liver using real-time PCR. Relative expression levels of IRF3 mRNA were detected in liver after normalization to GAPDH through real-time PCR. Data represent means ± SD (n = 6 each group). **C**. IRF3 protein expression in liver using Western blot. Left panel shows a representative picture of Western blot, and right shows relative levels of IRF3 protein after normalization to GAPDH. Data represent means ± SD (n = 6 each group). *P<0.05 and **P<0.01 versus control mice [urantide(-)LPS/D-GalN(-)]; #*P*<0.05 and ##*P*<0.01 versus LPS/D-GalN-challenged mice [urantide(-)LPS/D-GalN(+)]. M, DL2000 DNA marker.

In this experiment, we used our previously constructed ALF mouse model [16] to investigate IRF3 expression in the livers of the mice. The reverse transcription PCR and real-time PCR results both showed that IRF3 mRNA expression in liver tissues was significantly increased in the ALF mice. After pre-treatment with the UT receptor antagonist urantide, the IRF3 mRNA level induced by LPS/D-GalN was significantly decreased (Figure 1A and 1B). The liver IRF3 protein expression level in the ALF mice showed the same trend. As shown in Figure 1C, urantide inhibited the IRF3 protein expression induced by LPS/D-GalN. These results suggest that the UII/UT signaling system mediates the LPS/D-GalN-induced up-regulation of IRF3 expression in liver tissues in ALF mice.

### Urantide inhibited IRF3 gene transcription and protein nuclear translocation in LPS-stimulated KCs

**Figure 2:**
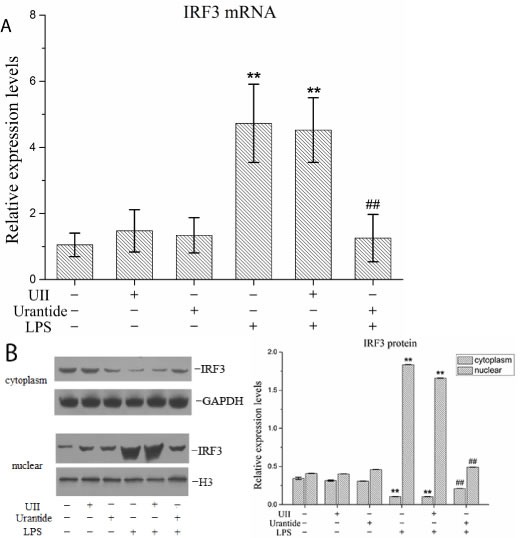
urantide inhibits IRF3 expression and activation in LPS-stimulated KCs KCs were treated with UII or urantide 0.5 h before LPS stimulation. **A**. IRF3 mRNA expression in KCs using real-time PCR. Relative expression levels of IRF3 mRNA were detected in KCs after normalization to GAPDH through real-time PCR. Data represent means ± SD (n = 6). **B**. IRF3 protein expression in the nuclear and cytoplasm of KCs using Western blot. Left panel shows representative pictures of Western blot, and right shows relative levels of nuclear and cytoplasm IRF3 protein after normalization to H3. Data represent means ± SD (n = 6). *P<0.05 and **P<0.01 versus control cells [UII(-)urantide(-)LPS(-)]; #*P*<0.05 and ##*P*<0.01 versus LPS-stimulated cells [UII(-)urantide(-)LPS(+)].

The primary KC isolation, culture, and identification results were shown in our previous article [20]. To analyze the function of the UII/UT system in liver IRF3 expression, we performed primary KC isolation and culture techniques in this experiment to analyze the effect of the UII/UT system on IRF3 expression under LPS stimulation. Our previous studies confirmed that the expression of UII and its receptor UT was significantly increased after the stimulation of primary KCs with LPS [20]. In this experiment, we pretreated the KCs with UII or urantide to assess IRF3 expression after LPS stimulation. The IRF3 mRNA expression levels in the KCs are shown in Figure 2A. LPS stimulation induced a significant up-regulation of the cellular IRF3 mRNA levels, which was inhibited by urantide pre-treatment. In contrast, UII did not have a significant effect on IRF3 mRNA expression. Cytoplasmic and nuclear IRF3 protein expression in KCs is shown in Figure 2B. LPS stimulation increased the IRF3 protein levels in the KC nuclei and significantly decreased the IRF3 protein levels in the cytoplasm. Urantide pre-treatment inhibited the increase in the nuclear IRF3 levels and the down-regulation of the cytoplasmic IRF3 levels after LPS stimulation. Application of UII did not have a significant effect on the IRF3 protein levels in the KC cytoplasm or nuclei. These results suggested that LPS induced IRF3 gene transcription and protein nuclear translocation in KCs and that urantide inhibited the stimulatory effect of LPS, indicating that LPS-induced IRF3 expression and activation depended on UII/UT signal transduction.

### Urantide inhibited IFN-β, IL-6, and IFN-γ cytokine secretion from LPS-stimulated KCs

**Figure 3:**
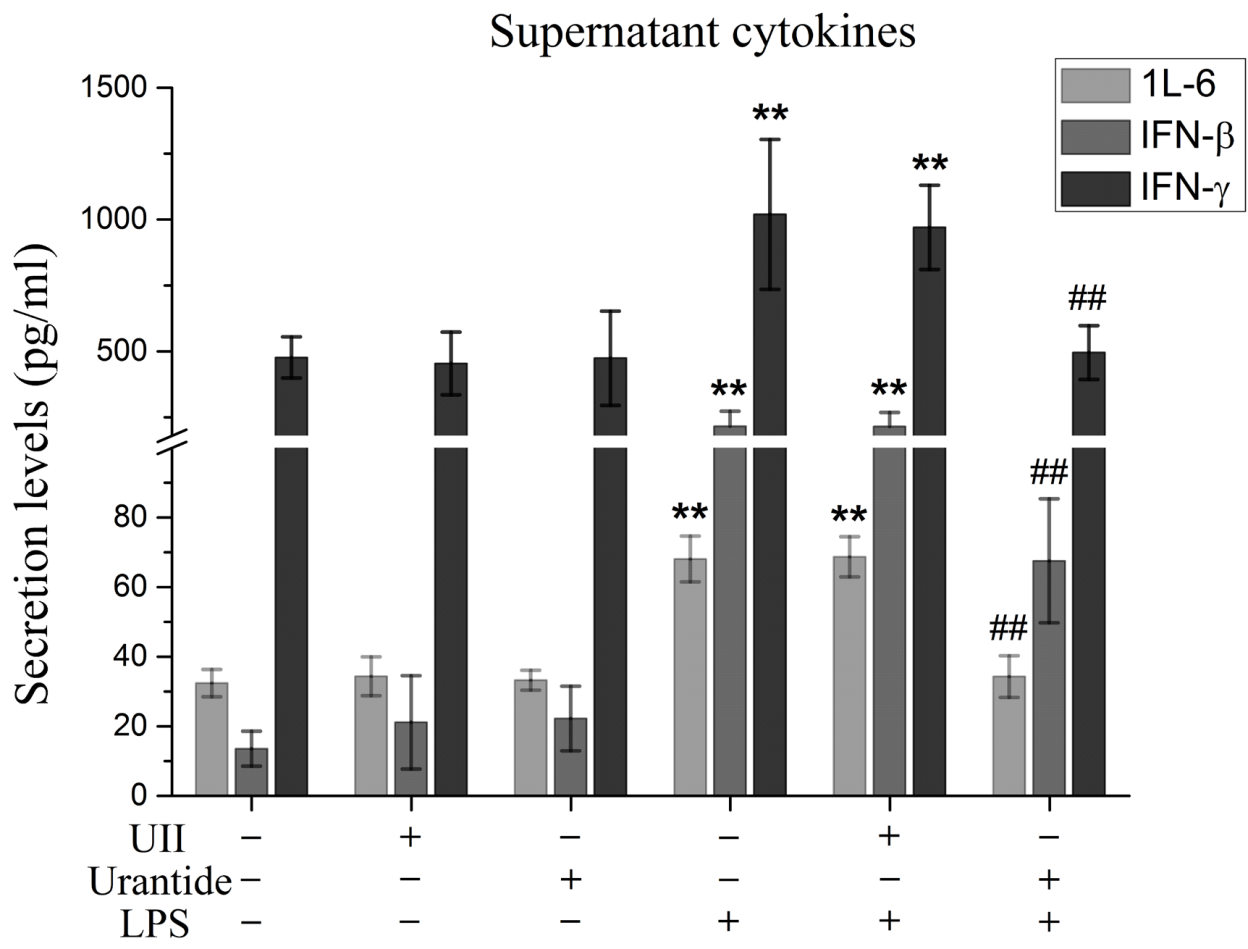
urantide inhibits secretions of IFN-β, IL-6 and IFN-γ in LPS-stimulated KCs KCs were treated with UII or urantide 0.5 h before LPS stimulation. Secretion levels of IFN-β, IL-6 and IFN-γ in KCs. Culture supernatant were assayed for IFN-β, IL-6 and IFN-γ via ELISA. Data represent means ± SD (n = 6). *P<0.05 and **P<0.01 versus control cells [UII(-)urantide(-)LPS(-)]; #*P*<0.05 and ##*P*<0.01 versus LPS-stimulated cells [UII(-)urantide(-)LPS(+)].

In this experiment, we evaluated the secretion of the IRF3 downstream molecule IFN-β and the NF-κB/MAPK downstream molecules IL-6 and IFN-γ. Our results showed that IL-6, IFN-β, and IFN-γ secretion from KCs was significantly increased after LPS stimulation and significantly inhibited by urantide pre-treatment of the LPS-stimulated KCs (Figure 3). UII application did not affect the secretion of these cytokines from the KCs. These results suggested that the UII/UT system had activation functions in both IRF3 innate immune signaling and the NF-κB/MAPK inflammatory signaling pathways.

### The IRF3 shRNA adenovirus knocked down the IRF3 levels in LPS-stimulated KCs

**Figure 4:**
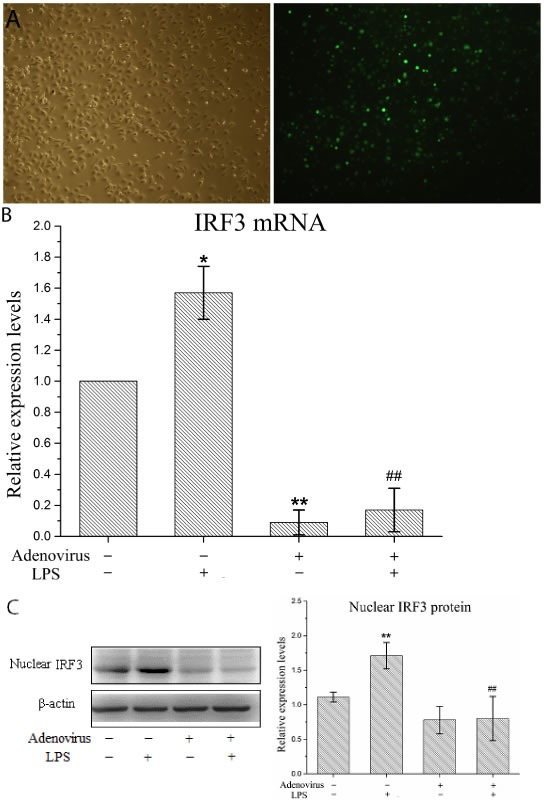
Interference adenovirus knockdowns IRF3 expression in LPS-stimulated KCs KCs were infected with IRF3 shRNA adenovirus 48 h before LPS stimulation. **A**. Manifestation of KCs after infected with adenovirus under microscopy. Left panel shows a representative picture of KC's morphology in light microscopy, and right shows GFP expression of KCs using fluorescence microscopy. **B**. IRF3 mRNA expression in KCs using real-time PCR. Relative expression levels of IRF3 mRNA were detected in KCs after normalization to GAPDH through real-time PCR. Data represent means ± SD (n = 6). **C**. IRF3 protein expression in the nuclear of KCs using Western blot. Left panel shows a representative picture of Western blot, and right shows relative levels of nuclear IRF3 protein after normalization to β-actin. Data represent means ± SD (n = 6). *P<0.05 and **P<0.01 versus control cells [adenovirus(-)LPS(-)]; #*P*<0.05 and ##*P*<0.01 versus LPS-stimulated cells [adenovirus(-)LPS(+)].

We performed gene interference experiments in KCs. As shown in Figure 4A, the infection efficiency of the IRF3 shRNA-carrying adenovirus in the KCs reached 100%. Next, we detected the gene silencing efficacy of this virus in the KCs. We showed that the IRF3 shRNA significantly inhibited the constitutive expression of the IRF3 mRNA in resting (non-LPS stimulated) KCs and the induced IRF3 mRNA expression in LPS-stimulated KCs (Figure 4B). Additionally, the shRNA inhibited IRF3 protein expression in the nuclei of LPS-stimulated KCs but did not have an effect on constitutive nuclear IRF3 expression in the KCs (Figure 4C). These results suggested that our interference adenovirus construct effectively knocked down IRF3 gene expression and reduced the level of functional IRF3 protein expression in the nuclei of LPS-stimulated KCs.

### IRF3 functions in the inflammatory response in KCs

**Figure 5:**
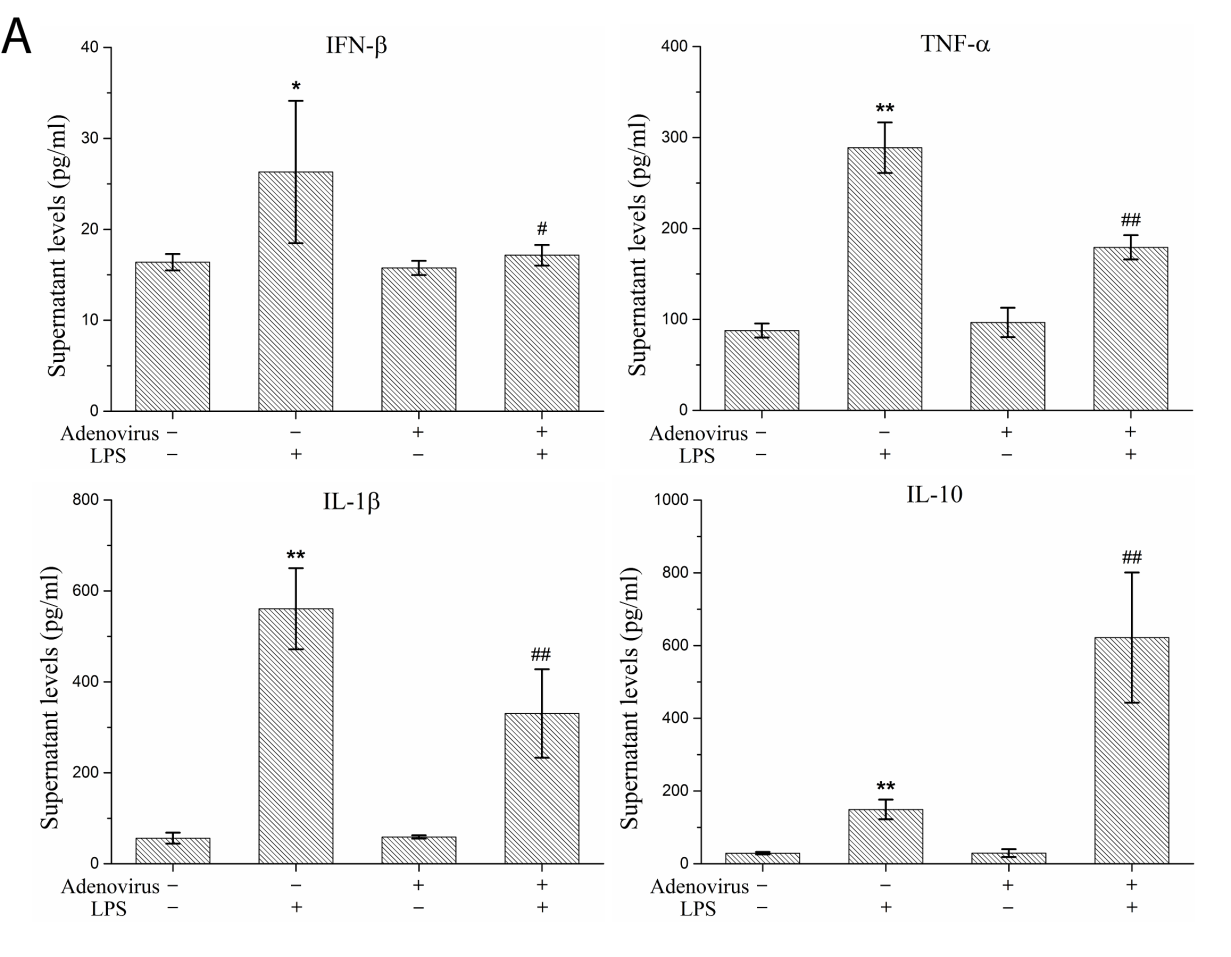

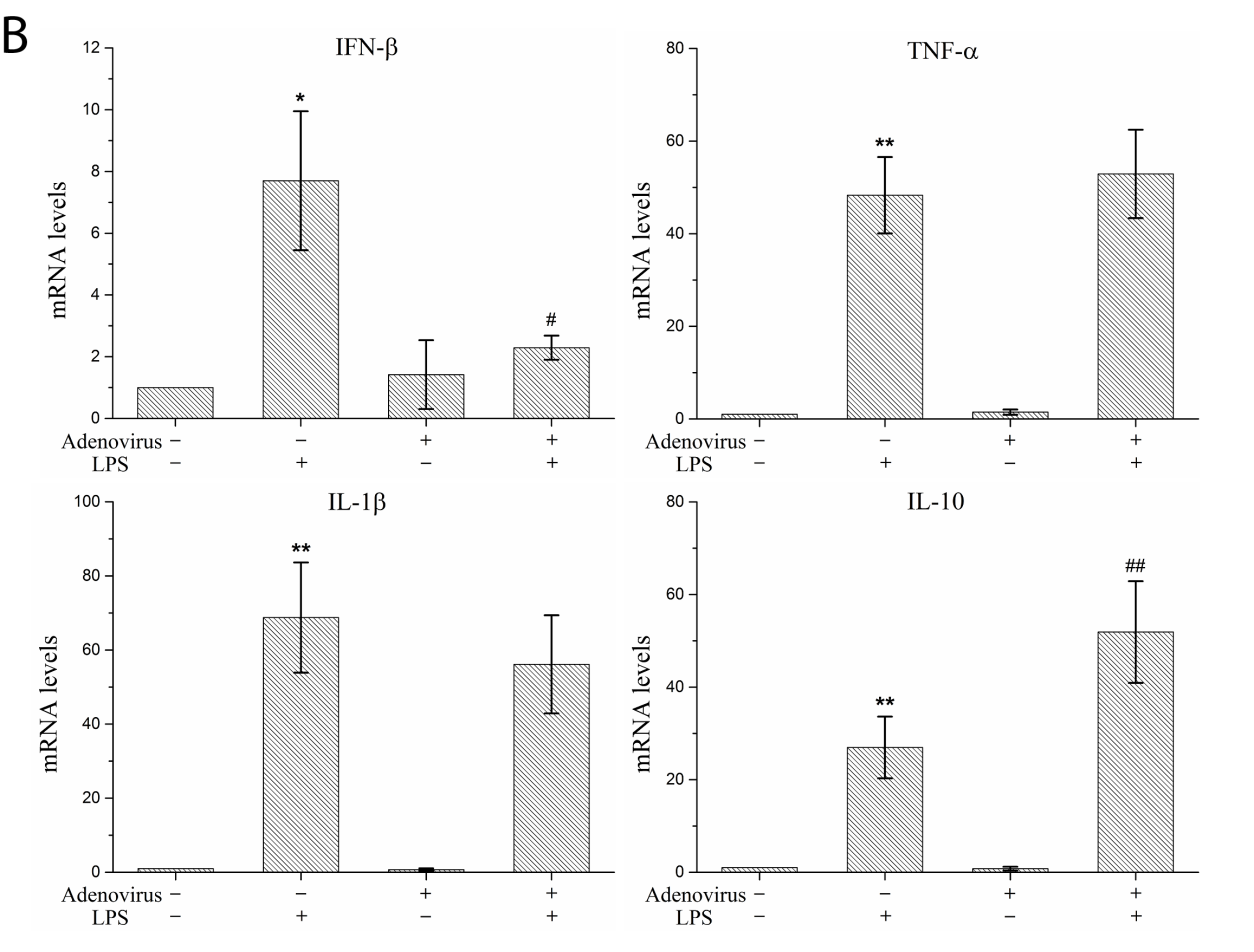

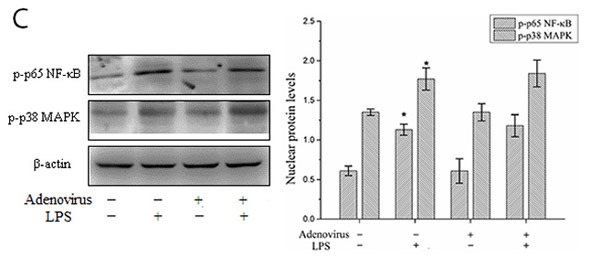
Effect of IRF3 on immune inflammatory responses in KCs KCs were infected with IRF3 shRNA adenovirus 48 h before LPS stimulation. **A**. Secretion levels of IFN-β, TNF-α, IL-1β and IL-10 in KCs. Culture supernatant were assayed for IFN-β, TNF-α, IL-1β and IL-10 via ELISA. Data represent means ± SD (n = 6). **B**. mRNA expression of IFN-β, TNF-α, IL-1β and IL-10 in KCs using real-time PCR. Relative expression levels of IFN-β, TNF-α, IL-1β and IL-10 mRNA were detected in KCs after normalization to GAPDH through real-time PCR. Data represent means ± SD (n = 6). **C**. p-p65 and p-p38 MAPK protein expressions in the nuclear of KCs using Western blot. Left panel shows representative pictures of Western blot, and right shows relative levels of nuclear p-p65 and p-p38 MAPK protein after normalization to β-actin. Data represent means ± SD (n = 6). *P<0.05 and **P<0.01 versus control cells [adenovirus(-)LPS(-)]; #*P*<0.05 and ##*P*<0.01 versus LPS-stimulated cells [adenovirus(-)LPS(+)].

To analyze the inflammatory effects of IRF3, we examined the expression and secretion of IFN-β, TNF-α, IL-1β, and IL-10 in LPS-stimulated KCs based on the gene expression silencing studies. The results showed that the IRF3 shRNA significantly inhibited IFN-β mRNA expression and protein secretion and TNF-α and IL-1β protein secretion in LPS-stimulated KCs. Additionally, IRF3 knockdown promoted IL-10 mRNA expression and protein secretion. However, the IRF3 shRNA did not inhibit TNF-α and IL-1β mRNA expression in the LPS-stimulated KCs and did not have a significant effect on the constitutive gene expression and secretion of IFN-β, TNF-α, IL-1β, and IL-10 in resting KCs (Figure 5A and 5B). We also detected the phosphorylation levels of the NF-κB p65 protein (p-p65) and p38 MAPK protein (p-p38 MAPK) in the KC nuclei. The results showed that the IRF3 shRNA adenovirus did not have significant effects on nuclear p-p65 and p-p38 MAPK protein expression in the LPS-stimulated KCs and their constitutive expression in the cells (Figure 5C).

## DISCUSSION

Studies have shown that liver UII/UT expression is increased in ALF patients [15] and that the UII/UT system mediates inflammatory injury of the liver tissues in ALF [16]; however, the mechanism underlying the acute inflammatory injury of liver tissues mediated by the UII/UT system has not been completely elucidated. In this study, we showed that transcription factor IRF3 expression was significantly up-regulated in the livers of ALF mice. IRF3 participates in the immune liver injury induced by HCV infection [27, 28] and plays an important role in the development of alcoholic liver injury and ischemia/reperfusion liver injury [29, 30]. IRF3 knockout protects experimental rabbits from the development of alcohol-induced hepatic steatosis, alcoholic hepatitis, and liver injury [30]. Here, for the first time, liver IRF3 was reported to be up-regulated in ALF. This result suggested that IRF3 might participate in inflammatory liver tissue injury in ALF similar to the UII/UT signaling system. In this study, we showed that the UT-specific antagonist urantide inhibited liver IRF3 expression in ALF. This result indicated that liver IRF3 expression in ALF depended (at least partially) on UII/UT system signal transduction; in other words, IRF3 might participate in the inflammatory injury mechanism of the UII/UT system.

To investigate the relationship between the UII/UT system and the IRF3 molecule in the inflammatory process, we performed experiments with primary KCs to analyze the effect of the UII/UT system on IRF3 expression in KCs under LPS stimulation. Stimulation of KCs by LPS significantly up-regulated the expression of UII and its receptor UT [20]. In this study, we showed that LPS also induced IRF3 mRNA expression in KCs and that the IRF3 cytoplasmic protein level in KCs was decreased after LPS stimulation. We also assessed the IRF3 protein expression levels in the KC nuclei and found that LPS stimulation resulted in a significant increase in the IRF3 protein levels. These results indicated that LPS induced IRF3 gene transcription and promoted the nuclear translocation of the IRF3 protein. Studies confirmed that IRF3 was primarily present in the cytoplasm in an inactive form in resting cells. After stimulation of the cells by inflammation, IRF3 was activated and translocated into the nucleus. In the nucleus, IRF3 induced target gene transcription to exert its biological effects [31, 32]. We showed that the application of urantide significantly inhibited the IRF3 transcription and nuclear translocation induced by LPS. These results indicated that the induction of IRF3 expression and activation in KCs by LPS required the UII/UT signaling system. Therefore, the UII/UT signaling system mediated IRF3 expression and activation in KCs following LPS stimulation. Our experiments also showed that UII application alone did not have a positive regulatory effect on IRF3 expression and nuclear translocation in KCs and that the combined application of UII and LPS did not have the expected additive effects. One possible reason might be the low UT expression level in non-activated (or resting) KCs. Additionally, studies confirmed that UT was expressed in the cell membrane [3] and a large amount of UT was present in the nuclear membrane [33] after the cells were stimulated by inflammation. Therefore, the intracellular UII/UT signaling pathway may play more important roles in IRF3 expression and activity in LPS-stimulated KCs.

To explain the influence of UII/UT on the innate immunity and inflammatory activation effects in KCs, we evaluated the secretion of IFN-β, which is downstream of IRF3 signaling, and IL-6 and IFN-γ, which are downstream of NF-κB/MAPK signaling. We showed that IL-6, IFN-β, and IFN-γ secretion in KCs was significantly increased after LPS stimulation, whereas urantide application significantly inhibited the stimulatory effect of LPS in KCs. These results indicated that the UII/UT system had important positive regulatory effects on both the IRF3 innate immune signaling pathway and the NF-κB/MAPK inflammatory signaling pathway. Therefore, the innate immune and inflammatory activities are activated in LPS-stimulated KCs due to the unity in the functions of the UII/UT system. Because IRF3 is a critical molecule in the innate immune signaling pathway, it is not clear whether IRF3 also has inflammatory activation effects.

To explore IRF3 functions in the inflammatory reaction, we used the IRF3 shRNA adenovirus (interference adenovirus) to perform IRF3 gene knockdown or interference experiments. We showed that the interference adenovirus infected primary KCs at a high efficiency and effectively inhibited IRF3 mRNA expression in the cells. Studies on IRF3 protein expression showed that the shRNA significantly inhibited IRF3 protein expression in the nuclei of LPS-stimulated KCs but did not affect the constitutive IRF3 protein expression level in the nuclei of non-LPS-stimulated KCs (i.e., resting state KCs). These results may be associated with the inhibition of nuclear translocation or nuclear trafficking because the IRF3 protein is in a non-active state in resting KCs.

Precise cellular localization is very important for the function of most signaling proteins. For instance, nuclear translocation is the first step for transcription factors to exert their transcription regulatory effects [34]. IRF3 is an important transcription factor in cells. Its regulation of cell functions depends on the nuclear import level of this protein [35]. In this study, we found that the expression and secretion of the cytokine IFN-β was decreased with the down-regulation of the IRF3 protein level in the nucleus after IRF3 gene silencing in KCs by the interference adenovirus. These results indicated that the interference adenovirus effectively blocked cellular IRF3 signal transduction. Additionally, the TNF-α and IL-1β concentrations in the supernatants were down-regulated, but TNF-α and IL-1β mRNA expression in the cells was not affected. These results suggested that IRF3 promoted TNF-α and IL-1β secretion but did not affect the transcription of these genes in the cells. Therefore, IRF3 might play a role in the post-transcriptional regulation of the pro-inflammatory cytokines TNF-α and IL-1β. The cytokine IFN-β induces TNF-α and IL-6 secretion (but not mRNA expression) in LPS-stimulated macrophages through the activation of the downstream interferon-stimulated genes (ISGs) [36]. However, additional studies showed that the production of the above effects depended on the activation of IRF3 by ISGs [36]. These results indicated that IRF3-IFN-β signal transduction in LPS-stimulated KCs or macrophages might have autocrine regulatory effects. The innate antimicrobial immune effects of IRF3 might be strengthened through this positive feedback autocrine regulation and the inflammatory secretion effects on the cells might exhibit cascade amplification. Thus, IRF3-IFN-β signaling not only induces the innate immune responses in cells to pathogenic microbes [34] but also plays important roles in the occurrence and development of inflammatory tissue injury [37, 38].

In this study, we found that LPS-induced IL-10 expression and secretion were significantly increased after IRF3 gene silencing. This result suggested that IRF3 inhibited IL-10 expression and secretion in LPS-stimulated KCs. IL-10 is an anti-inflammatory cytokine with immune regulatory effects that can induce SOCS3 expression to inhibit the expression of the inflammatory cytokines TNF-α, IL-6, and iNOS in LPS-stimulated macrophages [39]. A recent report showed that IL-10 degraded the NF-κB p65 subunit and induced MAPK phosphatases to inactivate MAPK signaling [40]. Additionally, IL-10 inhibited macrophage activation and inhibited the immune response of innate immune cells to pathogens [41]. Therefore, IL-10 might be a negative feedback regulatory molecule in TLR4 inflammatory signal transduction. During injury stimulation, the inhibitory effects of IRF3 on the IL-10 overexpression induced by LPS could help increase the cellular innate immune response and inflammatory response to tissue injury.

To investigate the effect of IRF3 on the MyD88-dependent signaling pathway, we studied the NF-κB p65 subunit and p38 MAPK protein phosphorylation levels in the KC nuclei. LPS stimulation induced phosphorylated NF-κB and p38 MAPK protein expression in KCs. After IRF3 gene silencing by application of the interference adenovirus, the activated NF-κB and p38 MAPK protein levels in the nuclei were not significantly changed in the LPS-stimulated KCs. These results indicated that activation of the transcription factor IRF3 in LPS-stimulated KCs did not influence the status of the nuclear NF-κB and p38 MAPK activities.

In summary, this study, for the first time, revealed the functions of IRF3 in ALF mediated by the UII/UT system. Through the IRF3 functions, the UII/UT system initiated innate immunity and the inflammatory response, thereby releasing signals in KCs to achieve real organic unity between the immune responses and inflammatory injury effects in the liver. This study also elucidated the molecular mechanism underlying ALF mediated by the UII/UT system. In this mechanism, the UII/UT system not only activated the MyD88-dependent NF-κB and p38 MAPK pathways and promoted the expression and release of pro-inflammatory cytokines including TNF-α, IL-1β, and IL-6 but also induced the transcription and nuclear translocation of the IRF3 molecule via the Myd88-independent pathway. The functions of activated IRF3 might be even more complicated because IRF3 could influence many links in the inflammatory reaction in the liver. For example, IRF3 could activate the downstream IFN-β immune signaling pathway to promote the inflammatory release of TNF-α and IL-1β and inhibit the immune regulatory signaling of the anti-inflammatory cytokine IL-10. Therefore, IRF3 is an important molecule in inflammatory injury in ALF mediated by the UII/UT system.

## MATERIALS AND METHODS

### Materials

### Experimental animals

Clean healthy grade male Sprague Dawley (SD) rats with weights of 150 - 200 g and BALB/c mice with weights of 20 – 22 g, were provided by the Experimental Animal Center of the First People's Hospital affiliated with Shanghai Jiaotong University, and the Animal Certificate of Conformity number was SYXK (Shanghai) 2009-0086. The animals were maintained in specific pathogen free air at a temperature of 22±2°C with 12 h light and dark cycles and relative humidity of 50%. The animals had free access to water and food and were placed on 12:12 h light:dark cycles. The animals were fasted for 12 h before the experiments. Animals care and treatment were humanity and in compliance with the recommendations in the Guide for the Care and Use of Laboratory Animals of the National Institutes of Health. The protocol was approved by the Committee on the Ethics of Medical Scientific Research of the First People's Hospital, Shanghai Jiaotong University (Permit Number: 2012KY041). All surgery was performed under sodium pentobarbital anesthesia, and all efforts were made to minimize suffering.

### Main reagents and consumables

LPS (*Escherichia coli* strain O55: B5) and D-galactosamine (D-GalN) were obtained from Sigma–Aldrich (St.Louis, MO, USA). UII and Urantide were purchased from PEPTIDES (Japan). Antibodies against IRF3, phospho-NF-κB p65 (Ser536) and phospho-p38 MAPK (Thr180/Tyr182) were purchased from Cell Signaling Techology (Danvers, MA, USA). TRIzol and IV collagenase were purchased from Invitrogen (Carlsbad, CA, USA). 1640 medium and fetal bovine serum were purchased from Gibco (Carlsbad, CA, USA). PCR primers were synthesized by Shanghai Sangon Biotech (China). SYBR Green PCR kit were purchased from Thermo (Carlsbad, CA, USA). ELISA kit was purchased from eBioscience (San Diego, CA, USA). Protein extraction and Western blot kit were purchased from Pierce (Rockford, IL, USA).

### Methods

### Animal experimental

The experimental method was described in our previous article [16]. Briefly, Mice were pretreated before induction of ALF intravenously with a total volume of 100 μl of normal saline (NS) or with 0.6 mg.kg^−1^ urantide dissolved in 100 μl NS (urantide). At 30 min after the injection, mice (NS, urantide) were challenged by an intraperitoneal injection with a total volume of 200 μl of NS (sham) or with 800 mg.kg^−1^ GalN and 50 mg.kg^−1^ LPS dissolved in 200 μl of NS (LPS/GalN, urantide+LPS/GalN). The liver tissues were sampled at 12 h after LPS/GalN challenge.

### Isolation and culture of primary KCs

Rat KCs were isolated and cultured according to the method in our previous article [20]. In brief, rats were anesthetized via intraperitoneal injection of sodium pentobarbital (40 mg.kg^−1^) and received intraperitoneal injections of 1 mL of heparin. Disinfection was conducted by soaking in 75% alcohol. The abdomen was opened to reveal the portal vein. After ligation of the suprahepatic inferior vena cava, D-Hanks' solution at room temperature was slowly injected through the portal vein, and the hepatic inferior vena cava was cut open to allow blood and perfusion fluid to drain freely. After the liver volume showed enlargement and the color of various portions of the liver gradually turned white, the solution was switched to 0.5% IV collagenase digestion solution (prepared using Hank's balanced salt solution (HBSS)) to continue perfusion and digestion of the liver tissue. The liver was dissected and placed in IV collagenase digestion solution (containing 0.1% pronase E and 0.005% DNase I). The liver tissue was carefully torn apart and passed through a 200-mesh filter. The filtrate was centrifuged, and the pellet was subjected to discontinuous Percoll density gradient centrifugation. The membranous cell layer in the interface between 30% Percoll and 60% Percoll was carefully collected via aspiration. Cells were washed with HBSS, added to RPMI 1640 medium (containing fetal calf serum, penicillin, and streptomycin) and resuspended. After 1–2 h of incubation, non-adherent cells were washed away to obtain purified KCs. Phagocytosis testing using ink and ED2 staining was used to identificate the purified KCs and validate cell viability and purity. Upon cell purity > 90% and viability > 95%, cells were used for the next experiment.

### KC experimental

Primary KC treatment methods was described in our previous article [20]. Briefly, cells were seeded in 6-well plates with 4 × 10^6^ cells per well. After 24 h of incubation, cells were washed three times with phosphate buffered solution (PBS), and 500 μL of serum-free culture medium was added to each well, followed by pretreatment using UII or urantide solution (both were prepared in PBS). The UII final concentration was 100 nM, that of urantide was 20 nM, and the dosage and usage were applied as previously described [20] with minor modifications. After half an hour, LPS solution (20 μg.mL^−1^, prepared in PBS) was used to stimulate the cells. The final volume in each well was 515 μL. Six hours after LPS stimulation, cells and the culture supernatants were collected for use in subsequent experiments. The experiment was repeated six times.

### The IRF3 shRNA adenovirus and primary KC infection

The IRF3 shRNA green fluorescent protein (GFP)-expressing adenovirus was constructed by our laboratory and Obio Technology Co., Ltd. (Shanghai, China). The virus with the best inhibition efficiency (gene location 1219-1239: 5′- GGTTGTTCCTACATGTCTTAA-3′) in the inhibition efficiency studies was used for the experiments. This interference adenovirus was carried by the pAdeno-U6-CMV-EGFP plasmid (Genechem, Shanghai, China) and was constructed and packaged with the adenovirus backbone plasmid pBHGlox E1E3 (Biovector, Beijing, China). The GFP gene was used for convenient observation of the virus infection efficiency. After the construction of the interference adenovirus, primary KCs were seeded into 6-well plates (4×10^6^ cells/well). When the cells were stably attached after 24 h of culture, the culture supernatant was aspirated, and 500 μl of serum-free culture medium containing adenovirus at a multiplicity of infection (MOI) of 150 was added to each well; then, the cells were cultured for 2 h at 37°C in a 5% CO_2_ incubator. The supernatant was discarded and replaced with culture medium containing 5% serum for continuous culture. After 46 h, LPS at a final concentration of 20 μg.ml^−1^ was used to stimulate the cells according to the literature [20-26]. Cells and culture supernatants were collected after 6 h of LPS stimulation for the subsequent experiments.

### Reverse Transcription-polymerase Chain Reaction (RT-PCR)

Total RNA was extracted from liver tissues with TRIzol reagent following the manufacturer's instructions. The amount and integrity of RNA preparations was assessed using a spectrophotometer and an agarose gel electrophoresis. Two micrograms of total RNA were employed as the template for synthesis of first-strand cDNA with a M-MLV RT kit (Fermentas, Canada). The PCR primers were designed by Primer Premier 6.0 software (PremierBiosoft, PaloAlto, CA, USA). The primer sequences are described as Table 1. PCR was performed with the following thermal cycling conditions: denaturation at 94°C for 5 min followed by 32 cycles of denaturation at 94°C for 1 min, primer annealing at 51°C for 45 s and primer extension at 72°C for 45 s with a final extension at 72°C for 10 min. The PCR product were electrophed through 1.5% agarose gel stained with ethidium. The intensity of each product were analyied by the BIO-RAD Quantity-One 4.7 imaging system and normalized to the values for the housing keep gene Glyceraldehyde 3-phosphate dehydrogenase (GAPDH).

**Table 1:** Primer sequences used for RT-PCR

Genes		Primer sequences (5′→3′)	Product size (bp)
IRF3	Sense	5′ CGGAAAGAAGTGTTGCGGTTAG 3′	132
	Antisense	5′ TTTGCCATTGGTGTCAGGAGAG 3′	
GAPDH	Sense	5′ ATCACTGCCACCCAGAAG 3′	191
	Antisense	5′ TCCACGACGGACACATTG 3′	

### Real-time PCR

Extractions of total RNA and first-strand cDNA synthesis were performed as described in the RT-PCR methods. Primers were designed with Primer Premier 6.0 software, and the sequences and product lengths are shown in Table 2. Real-time PCR was performed according to kit instructions. The reaction system was supplemented with 2 × Premix Ex Taq^™^ II, sense and antisense strand primers, ROX Reference Dye II, and DNA templates and was amplified in the ABI 7500 instrument using the following two-step method: Step One: 50°C for 2 min, 1 cycle; Step Two: 95°C for 5 min, 40 cycles. GAPDH was used as the internal reference.

**Table 2:** Primer sequences used for real-time PCR

Genes		Primer sequences (5′→3′)	Product size (bp)
IRF3			
Primer1	Sense	5′ AGGAGCTGTTAGAGATGG 3′	103
	Antisense	5′ TACTGGTCAGAGGTAAGG 3′	
Primer2	Sense	5′ACGCACAGATGGCTGACTTT3′	102
	Antisense	5′TCCTCTTCCAGGTTGACAGG3′	
IFN-β	Sense	5′GCTGAATGGAAGGCTCAAC3′	105
	Anti-sense	5′TGAATGGCAAAGGCAGTGTA3′	
IL-1β	Sense	5′GCCAACAAGTGGTATTCTCCA3′	118
	Anti-sense	5′CCGTCTTTCATCACACAGGA3′	
TNF-α	Sense	5′CAGGTTCCGTCCCTCTCATA3′	100
	Anti-sense	5′TGCCAGTTCCACATCTCG3′	
GAPDH			
Primer1	Sense	5′ GTCGGTGTGAACGGATTTG 3′	181
	Antisense	5′ TCCCATTCTCAGCCTTGAC 3′	
Primer2	Sense	5′GACATGCCGCCTGGAGAAAC3′	92
	Antisense	5′GACATGCCGCCTGGAGAAAC3′	

### Enzyme-linked immunoassay (ELISA)

Cytokine levels in the cell supernatant were measured by double-antibody sandwich ELISA, and the procedures were performed in accordance with the kit instructions. In brief, the procedures are described as follows: samples or standards (100 μL.well-1) were added, followed by 90 min of incubation at 36°C; the plates were washed five times, and the biotinylated antibody solution (100 μL.well^−1^) was added, followed by 60 min of incubation at 36°C; the plates were washed five times and the enzyme conjugate working solution (100 μL.well^−1^) was added, followed by 30 min of incubation at 36°C in the dark; the plates were washed five times and the substrate (100 μL.well^−1^) was added, followed by 15 min of incubation at 36°C in the dark; the stop solution (100 μL.well^−1^) was added, and the OD450 values were measured after thorough mixing. The LPS or PBS treatment was applied to triplicate wells, and the results are presented as the mean of the triplicates.

### Extraction of nuclear and cytoplasmic protein

The procedures were conducted according to the instructions in the Pierce nuclear and cytoplasmic protein extraction kit. In brief, the following procedures were performed: cells were trypsinized and collected via centrifugation at 500 g for 5 min. The collected cells were washed twice with pre-chilled PBS, resuspended in CER I buffer at a 1:10 ratio by volume, and shaken vigorously for a few seconds for thorough re-suspension, followed by incubation on ice for 10 min. Ice-cold CER II buffer was added and thoroughly mixed, followed by incubation on ice for 1 min. The suspension was centrifuged at 16000 g for 5 min, the supernatant contained cytoplasmic protein and was collected. The pellet contained the nuclei. An equal volume of NER ice bath buffer was added to the pellet and mixed well, followed by incubation on ice for 40 min and centrifugation at 4°C and 16,000 g for 10 min. The supernatant represented the nuclear extract. UV spectrophotometry was used to determine protein concentration, and the extracts were subsequently aliquoted and stored at −80°C.

### Western-blot analysis

A total of 50 μg of protein was collected, boiled in Laemmli buffer for 10 min, and subjected to 10% sodium dodecyl sulfate/polyacrylamide (SDS-PAGE) electrophoresis, followed by transfer to the PVDF membrane. The PVDF membrane bound with proteins was blocked in 5% fat-free milk overnight at 4°C. After blocking, appropriate amounts of antibodies recognizing IRF3, p-p38, p-p65 and the internal reference were added, followed by incubation at room temperature for 2 h. The membrane was washed three times with 0.1% phosphate buffered saline Tween-20 (PBST) with 5 min per wash. Horseradish-peroxidase-labeled secondary antibodies were added, followed by incubation at room temperature for 1 h. The membrane was subsequently washed three times with 0.1% PBST with 5 min per wash. The results were detected with the ECL-Plus chemiluminescent detection kit, followed by X-ray film exposure. The results were observed after development and fixation of the film.

### Statistical analysis

The results were expressed as (mean ± SD) and intergroup comparison was performed using one-way analysis of variance (ANOVA). The SPSS13.0 statistical software was used for statistical analysis, and *P* <0.05 indicated a statistically significant difference.

## Abbreviations

UII: urotensin II
ALF: acute liver failure
KC: Kupffer cell
LPS: lipopolysaccharide
D-GalN: D-galactosamine
IRF3: interferon regulatory factor 3
TNF-α: tumor necrosis factor α
IL-1β: interleukin 1β
IL-6: interleukin-6
IL-10: interleukin-10
IFN-β: interferon-β
IFN-γ: interferon-γ
TLR4: toll-like receptor 4
MAPK: mitogen-activated protein kinase
NF-κB: nuclear factorκB
GFP: green fluorescent protein.
